# Impact of High-Intensity Sports Practice on Stomatognathic System Function: An Observational Study

**DOI:** 10.3390/dj13030126

**Published:** 2025-03-13

**Authors:** Evandro Marianetti Fioco, Marcelo Palinkas, Natália de Moraes Barbosa, Edson Donizetti Verri, Luciano Maia Alves Ferreira, Danilo Henrique Lattaro, Gabriella Simi Gariba Silva, Selma Siéssere, Simone Cecilio Hallak Regalo

**Affiliations:** 1 Department of Basic and Oral Biology, Ribeirão Preto School of Dentistry, University of São Paulo, Avenida do Café, s/n, Bairro Monte Alegre, Ribeirão Preto 14040-904, Brazil; evandroacm@claretiano.edu.br (E.M.F.); palinkas@usp.br (M.P.); natalia.mbarbosa@usp.br (N.d.M.B.); edverri@claretiano.edu.br (E.D.V.); lucianomaia@egasmoniz.edu.pt (L.M.A.F.); danilohl@usp.br (D.H.L.); gabriella.ssilva@sou.unaerp.edu.br (G.S.G.S.); selmas@forp.usp.br (S.S.); 2 Department of Physiotherapy, Claretiano Center University, São Paulo 14300-900, Brazil; 3 National Institute and Technology-Translational Medicine (INCT.TM), Ribeirão Preto 14040-904, Brazil; 4 Egas Moniz School of Health and Science, 2829-511 Almada, Portugal

**Keywords:** athletes, masticatory muscles, electromyography, ultrasonography, gnathodynamometry

## Abstract

**Background:** Physical activity improves quality of life, but competitive sports emphasize performance, leading to intense training and restrictive diets that increase injury risk. This affects the stomatognathic system, underscoring the role of sports dentistry in preventing injuries and orofacial functional changes. This observational study analyzed the stomatognathic system’s functionality in male high-intensity athletes (n = 18) compared to a sedentary group (n = 18). **Methods:** Functional parameters were analyzed: electromyographic activity during mandibular tasks, masseter and temporal muscles thickness, and molar bite force. **Results:** Student’s *t*-test was used for analysis. At mandibular rest, male high-performance athletes showed lower electromyographic activity in the right and left masseter muscles, suggesting adaptation to training. During maximum voluntary contraction, activity was higher in the temporal and masseter muscles, especially in the left masseter, indicating hypertrophy. Electromyographic activity increased during protrusion and lateral movements, particularly in the masseter and temporal muscles, demonstrating a greater functional efficiency group of athletes. They also exhibited greater masseter muscle thickness and thinner temporal muscle thickness, with a significant difference in the right masseter muscle at rest. The group of athletes showed greater molar bite force, with significant differences, indicating greater muscle capacity. **Conclusions:** Sports practice promotes adaptations in the stomatognathic system, improving its functionality.

## 1. Introduction

The search for quality of life encompasses aesthetics, health and well-being, which are enhanced by regular physical activity. However, in competitive sports, maximizing performance becomes a priority, requiring intense training, restrictive diets and increasing the risk of injury. These factors can undermine the benefits of physical activity, making an integrated approach to athlete health essential [1].

The stomatognathic system, composed of static and dynamic structures, is particularly affected by these demands. Sports dentistry has emerged as an essential field in the prevention and treatment of oral conditions that affect performance. Specialized intervention contributes not only to the treatment of injuries but also to the prevention of complications that can compromise athletic performance [2].

The relationship between dentistry and sport also extends to the prevention of muscle and joint injuries. Athletes often present changes in the stomatognathic system, such as increased bite force and hypertrophy of the masticatory muscles [3]. With aging, physiological changes occur, including bone and dental loss [4].

Each sport promotes specific adaptations in body posture. Understanding these particularities is essential for coaches, dentists and physical therapists, who must work together to optimize athletic performance [5]. As an example, at the 2019 Pan American Games in Lima, 76 of 6680 athletes required emergency dental care, with cavities and periodontal disease being the most common conditions identified [6]. Orofacial problems can lead to painful symptoms in the anatomical structures of the stomatognathic system and impair the athletic performance of elite athletes [7].

A review of oral trauma in elite athletes shows that sports such as basketball, hockey, and rugby have a high incidence of tooth fractures and facial injuries [8]. Lima and colleagues reviewed the prevalence of dental trauma in contact and non-contact sports, focusing on athletes over 18 years old. After analyzing 1707 articles, eight were selected, and three more were included after reviewing similar studies. The prevalence of dental trauma was 11.38% in contact sports and 5.24% in non-contact sports. The study concludes that the use of mouthguards is essential in both sports categories to prevent dental injuries [9]. Furthermore, factors such as carbohydrate intake and dry mouth during physical exertion contribute to the deterioration of oral health. Sevindik and colleagues analyzed oral health and cortisol levels in 91 winter sports athletes. Ice hockey players had more untreated cavities. There were no dental traumas, and dental erosion was lower in hockey and ski jumping. Cortisol levels varied across the sports, with biathlon having the lowest and ski jumping the highest. There was a negative relationship between salivary stress and oral health during the competitions [10]. Understanding these impacts can help improve athletes’ masticatory and respiratory function [11,12].

Sports dentistry has been consolidating itself as a fundamental discipline in maintaining oral health and optimizing physical performance [13]. Its integration with sports medicine allows the prevention and treatment of temporomandibular disorders and tooth wear, promoting better posture and reducing the risk of injuries [14]. Research continues to highlight how modifications to the stomatognathic system impact performance and how appropriate interventions can optimize athlete health and performance [15,16].

Therefore, the aim of this study was to evaluate the functionality of the stomatognathic system in male athletes who practice high-intensity activity, comparing them to a sedentary control group. For this purpose, the electromyographic activity and the thickness of the masseter and temporal muscles were analyzed, as well as the bite force of the molars. The hypothesis states that high-intensity athletes present an increase in electromyographic activity and thickness of the masseter and temporal muscles, in addition to maximal bite force.

## 2. Materials and Methods

### 2.1. Characterization of the Population and Sample

This prospective cohort observational study was approved by the research ethics committee of the School of Dentistry of Ribeirão Preto, University of São Paulo, Brazil (protocol code 60919222.2.0000.5419, date of approval: 3 August 2022). All subjects were previously informed about the study’s objectives and procedures, and they signed the informed consent form.

The sample size calculation was performed based on a pilot study involving 5 professional swimming athletes, using the software G*Power 3.1.9.2 (Franz Faul, Kiel University, Kiel, Germany). A significance level of α = 0.05, a 95% confidence interval, and a minimum statistical power of 80% were considered, which indicated the need for at least 10 subjects per group.

The sample consisted of 36 men, aged between 15 and 40 years. Of these, 18 were professional athletes for at least one year, forming the high-intensity male athlete group (age: 37.44 ± 4.14 years; weight: 88 ± 12.09 kg; height: 1.75 ± 0.08 m; BMI: 28.46 ± 2.62 kg/m^2^). The other 18 subjects were sedentary subjects, forming the control group (age: 39.05 ± 5.01 years; weight: 79.78 ± 10.22 kg; height: 1.78 ± 0.06 m; BMI: 25.16 ± 3.29 kg/m^2^).

The high training intensity of the athletes was defined by the fact that they had more than five years of continuous experience in the sport, training five times a week. However, the intensity of the workouts was not measured in a specific way; each athlete followed their usual training program, maintaining their regular exercise routine. The assessments were conducted during the athletes’ rest period, that is, at a time of low muscular demand.

The inclusion criteria required that subjects have natural dentition, including the presence of permanent first molars, normal occlusion, and no conditions that could affect the functionality of the stomatognathic system muscles. Exclusion criteria included the presence of ulcers, open wounds, skin hypersensitivity, cognitive impairment, neurological diseases, decompensated systemic conditions, a history of previous injuries, or prior orthodontic, physiotherapeutic, or speech therapy treatments. Also excluded were edentulous subjects, users of complete or removable dentures, subjects with active periodontal disease, and those using muscle relaxants that could alter neuromuscular physiology.

The exclusion of participants with conditions that affect the treatment response or modify oral function, such as systemic, neurological diseases, or specific oral conditions, aims to minimize biases that could distort the measurements. Furthermore, the exclusion of individuals with prior treatments or a history of injuries helps prevent these variables from interfering with the results, ensuring that the observed changes are attributed to the subject’s performance with or without training. These criteria help ensure that the data collected reflect the effects of the study more accurately.

### 2.2. Muscle Activity Analysis

The MyoSystem-I P84 electromyograph (MyosystemBr1, Data Hominis Tec. Ltda, Uberlândia, Minas Gerais, Brazil) was used to assess the electromyographic activity of the masseter and temporal muscles. Muscle activity in microvolts (µV) was analyzed during the following mandibular tasks: rest (4 s), maximal voluntary contraction dental clenching (10 s), protrusion (10 s), and right and left lateral movements (10 s each). Surface electrodes were positioned by a trained and qualified examiner, following the guidelines of the Superficial EMG for Non-Invasive Assessment of Muscles (SENIAM) Project [17]. The electromyographic signal acquisition was performed by calculating the root mean square [18]. Before the electrode placement, the skin was cleaned with alcohol to reduce impedance [19]. Additionally, the necessary instructions and explanations were provided, guiding the subjects to always remain calm.

### 2.3. Muscle Thickness Analysis

The assessment of the thickness of the ultrasonographic images of the masseter and temporal muscles was performed using the Nanomaxx ultrasound equipment (SonoSite, Inc., Bothell, WA, USA), equipped with a 13 MHz linear transducer. The images were acquired both in the mandibular rest position and during maximal voluntary contraction [20]. Each image capture was repeated three times, with 2 min intervals between each measurement in centimeters (cm), to calculate an average for data analysis. For the masseter muscle, the transducer was positioned approximately 1.5 to 2.0 cm above the mandibular angle toward the zygomatic arch. Regarding the temporal muscle, the transverse positioning of the linear transducer was performed on the belly of the temporal muscle, located in the temporal fossa region, approximately 1.0 to 1.5 cm backward and upward from the lateral commissure of the eyelids on both sides [21].

### 2.4. Molar Bite Force Analysis

Maximum molar bite force records were obtained using a digital dynamometer (Kratos, model IDDK, Cotia, SP, Brazil), with a maximum capacity of 1000 newtons (N) [20]. The equipment consisted of two rods, with the ends having Teflon disks that served as points for applying and recording the bite force. As a biosafety measure, the tips of the dynamometer were protected with disposable latex finger cots (Wariper-São Paulo, Brazil), following the guidelines of international studies. The dynamometer was placed on the region of the upper and lower permanent first molars, on both the right and left sides, to record three consecutive measurements, with a two-minute interval between each assessment. During the process, the subjects remained seated in a chair, with their arms relaxed by their sides and hands resting on their thighs, having been previously instructed and trained to correctly bite on the rods of the equipment, ensuring the accuracy of the results

### 2.5. Statistical Analysis

After data collection for the variables, the Shapiro–Wilk normality test was applied, which confirmed the normal distribution of the data. The statistical analysis was performed using SPSS software, version 22.0 (SPSS Inc., Chicago, IL, USA), employing the *t*-test to compare the groups regarding electromyographic activity, muscle thickness, and maximum molar bite force. The significance level adopted for the analyses was *p* < 0.05.

## 3. Results

Table 1 presents the electromyographic activity of the masseter and temporal muscles, comparing the results between the high-performance athlete group and the sedentary subjects group, considered as the control group. At mandibular rest, the high-performance male athletes showed lower electromyographic activity in the right masseter muscle (*p* = 0.000) and left masseter muscle (*p* = 0.046). During maximal voluntary contraction, activity was higher in the temporal and masseter muscles, especially in the left masseter (*p* = 0.011). Electromyographic activity increased during protrusion and lateral movements, especially for the masseter and temporal muscles, demonstrating greater functional efficiency in high-performance male athletes.

Table 2 shows the thickness of the masseter and temporal muscles and maximum molar bite force, comparing the results between the groups. In the high-performance male athletes, greater masseter muscle thickness and lower temporal muscle thickness were observed. A significant difference was found only in the right masseter muscle (*p* = 0.029) during mandibular rest, while no statistically significant differences were observed in the other muscles, either at rest or during maximum voluntary contraction. Additionally, the high-performance male athletes exhibited greater maximum molar bite force on both sides, with significant differences.

## 4. Discussion

The hypothesis was accepted because significant differences were found between the groups in electromyographic activity, the thickness of the masseter and temporal muscles, and maximum molar bite force. The results showed that the athlete group presented higher values in the variables analyzed. Regular physical activity is an essential factor for health and quality of life [22]. This study analyzed the functionality of the stomatognathic system in high-intensity athletes, and the results corroborate the existing literature, highlighting functional differences between athletes and sedentary individuals [23,24].

High-intensity male athletes showed lower electromyographic activity in the masseter muscles at rest, significantly differing from sedentary subjects. This finding may suggest adaptation to physical training, reducing basal muscle tone to minimize energy expenditure [25]. The reduction in basal muscle activity is in line with the expectation of minimal or no contraction at rest, with the mandibular balance being maintained by viscoelastic and proprioceptive factors [26]. However, some studies indicate residual electrical activity even at rest, highlighting the complexity of mandibular neuromuscular control [18,27].

During maximal voluntary contraction, higher electromyographic activity was observed in the temporal and masseter muscles, with particular emphasis on the left masseter, showing a significant difference when compared to the sedentary group. These results are in line with a previous study that points to hypertrophy of the masseter muscles in athletes [3], especially those who practice sports that require great muscular effort and load on the stomatognathic system.

Regarding mandibular protrusion and right and left lateral movements, the athletes exhibited higher electromyographic activity in the temporal and masseter muscles, with significant differences in both the temporal muscles and the left masseter compared to the sedentary subjects. These results reinforce the idea that regular physical training contributes to greater functional efficiency of the human body [28], resulting in likely better performance during mandibular movements. However, it is important to note that these adaptations, while necessary to meet the demands of sports, may also increase the risk of temporomandibular dysfunctions and related issues, such as muscle pain and headaches [29]. On the other hand, the sedentary subjects showed lower muscle activity, indicating a reduced functional use of the body muscles. This condition results in lower muscle tone and decreases functional efficiency when compared to high-performance athletes [30].

Studies indicate that intense physical training promotes neuromuscular adaptations, leading to greater motor unit recruitment and improved neural synchronization. These factors may explain the increased electromyographic activity observed in athletes [31,32]. Júnior et al. [1] analyzed masticatory efficiency, maximum bite force, and electromyographic activity of the masticatory muscles in upper-body strength training practitioners before and after exercise. The results showed a significant reduction in masticatory efficiency post-exercise, while bite force and electromyographic activity remained unchanged. Additionally, a positive correlation was observed between masticatory efficiency and strength.

The ultrasonographic analysis revealed a significant difference in the right masseter muscle thickness between the athletes and sedentary groups, with the athletes showing greater in the masseter muscles thickness and lower in the temporal muscle thickness. These results are consistent with the findings of previous studies that suggest physical activity can promote hypertrophy of skeletal striated muscles, adapting them to the functional demands of sports [33,34,35]. During maximal voluntary contraction, ultrasonography did not reveal significant differences in the masseter and temporal muscle thickness between the groups, but the pattern of lower thickness in the temporal muscles and greater thickness in the masseters was maintained in the athletes, reinforcing the idea that these changes are specific to sports training [36].

In terms of practical relevance, the increase of the masseter muscle thickness suggests an adaptation for activities requiring greater strength, which may be relevant in contact sports. The reduction in temporal muscle thickness may reflect an efficient adaptation to training. However, more studies are needed to understand how these differences directly impact athletic performance. From a clinical perspective, these adaptations may have implications for preventing muscle-related injuries, such as temporomandibular dysfunction.

Bite force results from the interaction between various components of the stomatognathic system, which are regulated by the central nervous system [37]. In this process, the action primarily occurs in the jaw elevator muscles and the skeletal and dental complex. Regarding maximum molar bite force, a significant difference was observed between the athletes and sedentary groups, with athletes demonstrating a greater ability to generate force, both on the right and left sides. This finding is consistent with studies that link physical training to increased overall muscle strength, including the masticatory muscles [38]. The practical relevance of this finding can be related to the impact of bite force on the athlete’s overall performance. Greater bite force may indicate better muscle control and efficiency, which can be important for jaw stability during sports movements that require greater endurance and functional control. However, it is important to highlight that the increase in bite force may be related to the development of habits, such as tooth clenching, a phenomenon frequently observed in athletes who face high levels of physical and emotional stress during competitions [30].

Increased load during sports activities can promote muscle hypertrophy and strengthen the stomatognathic system, contributing to greater molar bite force. Previous research indicates that athletes exhibit specific adaptations in the stomatognathic system, including changes in muscle morphology and functional efficiency, in response to the physical demands of training. In this context, Ribeiro et al. [39] assessed clinical signs of bruxism in CrossFit practitioners, observing behaviors such as teeth clenching, linea alba, and dental wear. Although no correlation was found between these signs and the intensity of physical activity, the results suggest that CrossFit^®^ practitioners may develop harmful oral habits that negatively impact the stomatognathic system, emphasizing the importance of oral health education for high-performance athletes.

The study presents findings that indicate functional adaptations of the stomatognathic system in high-intensity athletes, highlighting their implications for sports dentistry. These findings can contribute to the development of personalized preventive strategies, promoting athletes’ oral health. For example, the use of mouthguards helps reduce dental trauma and stress on the temporomandibular joint. Additionally, the muscle hypertrophy and increased bite force observed in the study suggest that athletes may be more susceptible to dental and restoration fractures due to this functional adaptation. Therefore, regular dental check-ups and interdisciplinary monitoring are essential for the early detection of dental fractures and other complications. Analyzing muscle adaptation patterns would also allow for occlusal adjustments and the application of specific relaxation therapies. Furthermore, longitudinal evaluations, dietary habits, and stress management should be integrated into athletes’ dental care to enhance both oral health and functional performance.

Sports dentistry plays an integral role in preventing and treating oral injuries and dysfunctions of the stomatognathic system. Interdisciplinary collaboration among dentists, physicians, and trainers is essential to optimize athletes’ functional performance. Since the observed muscular and functional adaptations can lead to consequences such as excessive dental wear, oral trauma, and temporomandibular dysfunction, continuous monitoring is indispensable for maintaining oral health and preventing potential complications.

The study has some limitations, such as the small sample size, consisting of 18 athletes and 18 sedentary subjects, which could impact the generalizability of the results. The male-only sample limits the generalizability of the findings, as sex-related differences in muscle physiology and bite force have been documented in the literature. Additionally, the study does not control external factors, such as dietary habits, stress, or oral health conditions, which may affect the stomatognathic system. Being cross-sectional, the study does not allow for the evaluation of changes over time, limiting the understanding of adaptations in the stomatognathic system in response to sports practice. The study does not take into account differences between sports disciplines. The type of sport could significantly influence the adaptation of the stomatognathic system (e.g., contact vs. non-contact sports). Future studies with larger samples and more controlled approaches are needed to confirm and deepen the conclusions.

## 5. Conclusions

This study concluded that male athletes who perform high-intensity activities present significant adaptations in the stomatognathic system, with differences in electromyographic activity, thickness of the masseter and temporal muscles, and maximum molar bite force compared to sedentary individuals. These changes may be a reflection of regular physical activity, improving the functional efficiency of the stomatognathic system.

## Figures and Tables

**Table 1:** Mean values and standard error of the electromyographic data between groups.

Mandibular Task Muscles (µV)	Groups		*p* Value	Cohen’s d	95%CI
	CG	AG			
	Mean ± Standard Error	Mean ± Standard Error			
Rest					
RM	6.73 ± 0.53	3.45 ± 0.24	0.000 *	1.82	1.04–2.59
LM	8.00 ± 0.71	5.97 ± 0.66	0.046 *	0.69	0.02–1.37
RT	7.77 ± 0.45	7.90 ± 1.65	0.944	0.03	−0.63–0.68
LT	8.56 ± 0.82	6.81 ± 1.01	0.189	0.45	−0.21–1.11
Protrusion					
RM	23.00 ± 4.27	35.52 ± 5.14	0.070	0.62	−0.04–1.29
LM	19.35 ± 3.53	46.68 ± 5.51	0.000 *	1.39	0.66–2.12
RT	8.94 ± 0.89	20.92 ± 2.83	0.000 *	1.34	0.62–2.07
LT	10.33 ± 1.40	20.37 ± 2.53	0.001 *	1.16	0.45–1.86
Right Laterality					
RM	8.14 ± 0.67	24.87 ± 2.23	0.000 *	2.39	1.53–3.25
LM	13.55 ± 2.14	24.47 ± 3.27	0.009 *	0.93	0.24–1.62
RT	12.67 ± 2.37	19.73 ± 4.03	0.141	0.50	−0.16–1.17
LT	9.92 ± 1.44	19.57 ± 3.12	0.143	0.94	0.25–1.62
Left Laterality					
RM	12.99 ± 1.55	20.42 ± 5.13	0.175	0.46	−0.20–1.12
LM	8.27 ± 0.57	34.17 ± 5.62	0.008 *	1.53	0.78–2.27
RT	9.15 ± 1.06	12.54 ± 2.59	0.234	0.40	−0.26–1.06
LT	12.79 ± 1.83	21.19 ± 2.28	0.000 *	0.96	0.27–1.65
MVC					
RM	105.79 ± 17.84	142.44 ± 28.71	0.286	0.36	−0.30–1.02
LM	97.96 ± 19.07	185.70 ± 26.75	0.011 *	0.89	0.21–1.57
RT	116.71 ± 20.14	170.13 ± 19.71	0.067	0.63	−0.04–1.30
LT	120.08 ± 15.66	199.34 ± 36.37	0.053	0.67	0.00–1.34

AG, High-performance male athletes; CG, sedentary control group; RM, right masseter; LM, left masseter; RT, right temporalis; LT, left temporalis; MVC, maximum voluntary contraction; µV, microvolts; CI, confidence intervals. Significant difference, student’s *t*-test (i.e., *p* < 0.05) *.

**Table 2:** Mean values and standard error of muscle thickness and bite force data between groups.

Variables	Groups		*p* Value	Cohen’s d	95%CI
	CG	AG			
	Mean ± Standard Error	Mean ± Standard Error			
Mandibular task Muscle thickness (cm)					
Rest					
RM	1.02 ± 0.03	1.19 ± 0.06	0.029 *	0.80	0.12–1.48
LM	1.06 ± 0.04	1.20 ± 0.05	0.055	0.68	0.01–1.35
RT	0.70 ± 0.02	0.64 ± 0.03	0.20	0.45	−0.21–1.11
LT	0.69 ± 0.03	0.64 ± 0.04	0.410	0.31	−0.35–0.97
MVC					
RM	1.35 ± 0.04	1.48 ± 0.06	0.122	0.56	−0.11–1.23
LM	1.44 ± 0.04	1.39 ± 0.07	0.533	0.19	−0.47–0.84
RT	0.79 ± 0.02	0.75 ± 0.05	0.509	0.24	−0.42–0.89
LT	0.80 ± 0.03	0.78 ± 0.05	0.689	0.10	−0.55–0.75
Molar bite force (N)					
Right	338.32 ± 34.51	662.42 ± 58.74	0.000 *	1.60	0.85–2.36
Left	381.86 ± 44.12	595.45 ± 59.62	0.003 *	0.96	0.27–1.65

AG, High-performance male athletes; CG, sedentary control group; RM, right masseter; LM, left masseter; RT, right temporalis; LT, left temporalis; MVC, maximum voluntary contraction.; cm, centimeters; N, newtons; CI, confidence intervals. Significant difference, student’s *t*-test (i.e., *p* < 0.05) *.

## Data Availability

The data presented in this study are available on reasonable request, after the signature of a formal data sharing agreement in anonymous form, from the corresponding author because they are protected by privacy.
